# Application of upright multidetector CT in orthopedics and the musculoskeletal system: insights into anatomy, alignment, and biomechanics

**DOI:** 10.1007/s11604-026-01999-3

**Published:** 2026-04-29

**Authors:** Yoshitake Yamada, Minoru Yamada, Yoichi Yokoyama, Kazuya Kaneda, Yuki Yoshida, Ryo Sasaki, Hiroyuki Seki, Ryo Mizukoshi, Fumiko Yagi, Noboru Matsumura, Taiki Nozaki, Takeo Nagura, Masaya Nakamura, Masahiro Jinzaki

**Affiliations:** 1 Department of Radiology, Keio University School of Medicine, 35 Shinanomachi, Shinjuku-ku, Tokyo 160-8582 Japan; 2 Department of Orthopaedic Surgery, Keio University School of Medicine, 35 Shinanomachi, Shinjuku-ku, Tokyo 160-8582 Japan; 3 Department of Orthopedic Surgery, Fussa Hospital, 1-6-1 Kamidaira, Fussa, Tokyo 197- 8511 Japan; 4 Department of Orthopaedic Surgery, NHO Tokyo Medical Center, 2-5-1 Higashigaoka, Meguro-ku, Tokyo 152-8902 Japan; 5 Department of Orthopaedic Surgery, Tachikawa Hospital, 4-2-22 Nishiki-cho, Tachikawa-shi, Tokyo 190-8531 Japan; 6 Department of Orthopedic Surgery, International University of Health and Welfare, Narita Hospital, 852 Hatakeda, Narita-shi, Chiba 286-8520 Japan; 7 Department of Radiology, National Defense Medical College, 3-2 Namiki, Tokorozawa, Saitama 359-8513 Japan

**Keywords:** Anatomy, Biomechanics, Multidetector computed tomography, Musculoskeletal diseases, Standing position, Weight-bearing

## Abstract

Upright multidetector CT (MDCT) enables whole-body, high-resolution, three-dimensional cross-sectional imaging in standing or sitting positions, providing detailed visualization of anatomy and biomechanics in physiologically relevant, load-bearing postures. This system maintains high spatial resolution and quantitative performance, including stable noise characteristics and consistent CT attenuation values, while improving workflow efficiency compared with conventional supine MDCT. In the shoulder and upper extremity, upright MDCT has clarified gravity-dependent alignment changes and enabled dynamic four-dimensional assessment of sternoclavicular and acromioclavicular joint motion. In the spine, it provides reproducible spinopelvic measurements, reveals posture-dependent narrowing of intervertebral foramina, and characterizes age- and sex-related differences in segmental spinal mobility. Upright MDCT of the knee allows precise evaluation of joint-line orientation, mechanical-axis migration, and rotational kinematics under weight-bearing conditions, enhancing diagnostic accuracy and preoperative planning in osteoarthritis. In the lower extremities, it captures kinetic-chain mechanics from the foot to the hip, integrating ground-reaction-force data to analyze load distribution and compensatory motion across adjacent joints. Upright MDCT also visualizes thoracic cage deformation and vertebral displacement in conditions such as pectus excavatum, demonstrating load-related changes that supine imaging may overlook. This review provides a state-of-the-art overview of upright MDCT applications in orthopedic imaging and highlights its potential to transform diagnostic strategies and deepen understanding of musculoskeletal pathophysiology under physiologically relevant weight-bearing conditions. Collectively, these studies demonstrate that upright MDCT is a quantitatively robust modality that offers new insights into functional anatomy, disease mechanisms, and biomechanical adaptation. By bridging diagnostic imaging with functional assessment, upright MDCT has the potential to refine understanding of musculoskeletal pathophysiology and to guide more personalized therapeutic strategies in orthopedic and related fields.

**Supplementary Information:**

The online version contains supplementary material available at 10.1007/s11604-026-01999-3.

## Introduction

Computed tomography (CT), developed in the late 1960s and widely implemented since the early 1970s [1], has been a cornerstone of diagnostic imaging for over half a century [2]. Traditionally performed in the supine position, CT has contributed to the detection and staging of major diseases, including cancer, arteriosclerosis, and infections [3–7]. The advent of multidetector-row CT (MDCT) around 2000 improved spatial and temporal resolution and enabled rapid acquisition of high-quality images [2, 3, 8, 9].

As societies worldwide confront super-aging populations, extending not only life expectancy but also healthy life expectancy has become a major healthcare priority [10, 11]. This requires early detection of functional impairments, many of which manifest under weight-bearing conditions [12, 13]. However, although most daily activities are performed in upright postures, almost all CT and MRI examinations are conducted in the supine position, limiting the evaluation of posture-dependent anatomical and physiological changes relevant to functional health.

To acquire three-dimensional images in a standing posture, cone-beam CT (CBCT) and upright MRI have been available. However, both modalities exhibit inherent technical limitations: CBCT is affected by slow gantry rotation speeds, lower contrast resolution, and a limited field of view [13–15], whereas upright MRI commonly operates at low magnetic field strengths (0.2–0.6 T), resulting in prolonged scan times, motion artifacts, and reduced image quality compared with conventional MRI [16, 17]. Consequently, their applicability for comprehensive whole-body evaluation remains restricted [18–20].

**Fig. 1:**
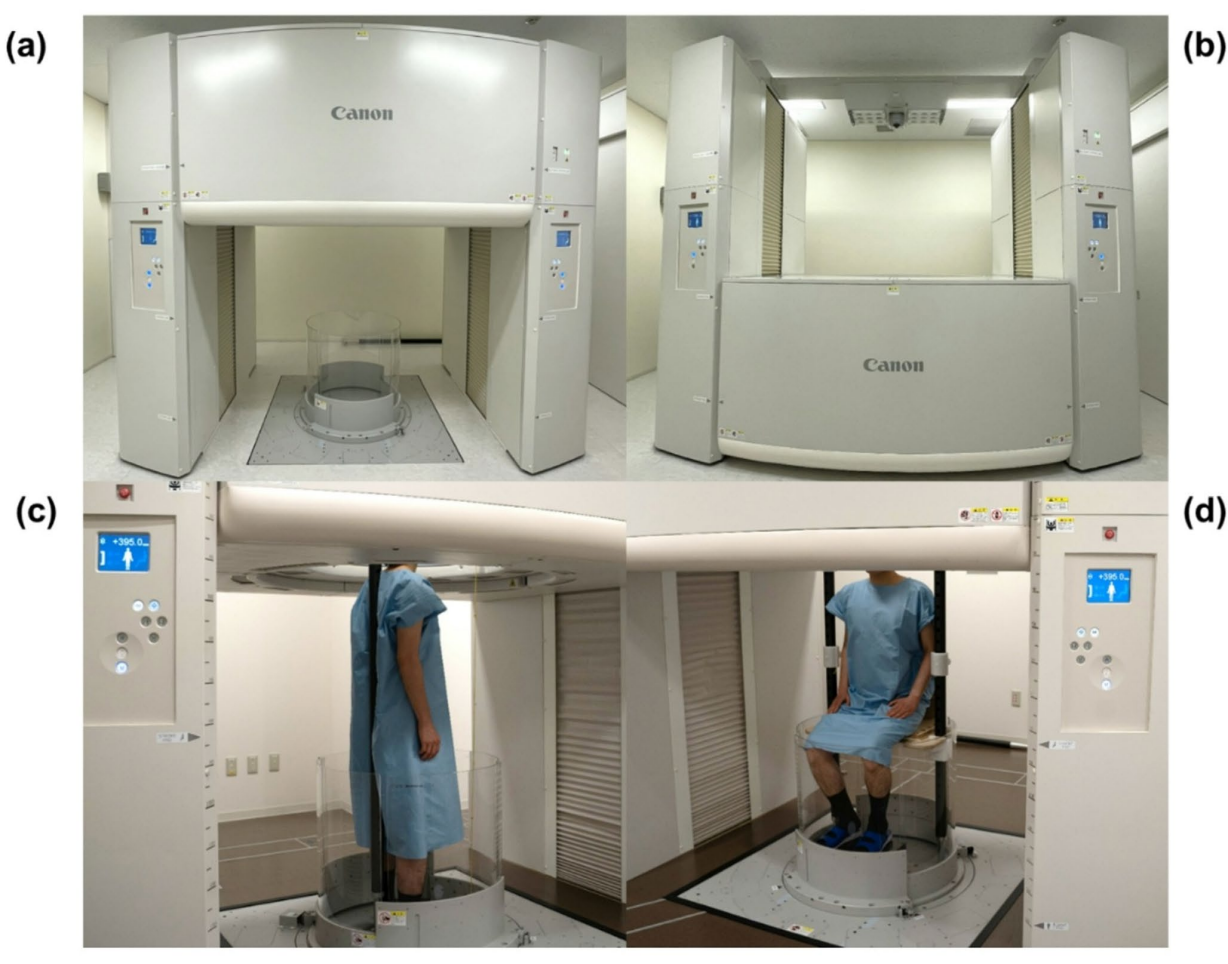
Upright multidetector CT (MDCT) system and safety-support devices. The upright MDCT system enables vertical movement of a transverse 320-detector-row gantry to perform imaging in multiple upright postures. **a** Gantry positioned at the upper end. **b** Gantry positioned at the lower end. **c** Standing-position imaging setup showing the support pole that provides gentle posterior stabilization and the knee-height transparent acrylic barrier that prevents falls. **d** Sitting-position imaging setup

In contrast, with the advent of 64- and 80-detector-row CT scanners, MDCT has enabled imaging of the body trunk in less than 20 s [18, 19]. This acquisition time approximates how long most individuals can comfortably maintain a standing posture, thereby making upright MDCT clinically feasible [18, 19]. In this context, Jinzaki and colleagues proposed the concept of a whole-body upright MDCT system to Canon Medical Systems (formerly Toshiba Medical Systems). The project was subsequently approved, and an upright MDCT system was eventually developed through an academic–industrial collaboration [18, 19]. This upright MDCT system preserves the high spatial resolution (0.5 mm detector size), rapid gantry rotation (0.275 s), and quantitative performance of conventional 320-detector-row CT while enabling imaging in physiologically relevant standing and sitting positions (Fig. 1a–d) [18]. The system maintains comparable image quality, including noise characteristics, spatial resolution, and CT attenuation value, improves clinical workflow, and reduces total examination access time by more than half compared with conventional supine MDCT [18]. Furthermore, upright MDCT enables remote operation and independent patient positioning, which may enhance safety and operational efficiency, particularly in infectious disease settings [18, 19].

Recent studies have shown that gravitational effects in the upright position alter the morphology and spatial relationships of organ systems, including the brain, lungs, airways, pelvic floor, vasculature, and musculoskeletal structures [18–27], underscoring the importance of posture-aware imaging. Among these, orthopedic imaging may benefit particularly from upright MDCT, which enables precise three-dimensional assessment of load-dependent anatomical changes, joint alignment, and biomechanical function.

This review provides a state-of-the-art overview of upright MDCT applications in orthopedic imaging and highlights its potential to transform diagnostic strategies and deepen understanding of musculoskeletal pathophysiology under physiologically relevant weight-bearing conditions.

### Constitution of the upright MDCT system

The upright MDCT system is designed to enable vertical movement of a transverse 320-detector-row gantry (detector element size, 0.5 mm). The gantry rotates at a maximum speed of 0.275 s per revolution and acquires up to 1200 projections per rotation, providing high temporal and spatial resolution (Fig. 1a–d) [18, 19]. This system supports imaging in either 80-detector-row or 320-detector-row acquisition modes. The gantry is mounted on dual lateral stands that ensure structural rigidity and stability during operation. Table 1 summarizes the principal technical specifications and system configurations of the conventional and upright 320-detector-row CT scanners [19]. Although both systems share nearly identical hardware specifications, their overall configurations differ substantially. The most notable differences involve imaging posture, supine in the conventional scanner and standing or seated in the new system, and installation footprint, namely the physical floor space required for installation, which is reduced to approximately two-thirds in the upright system. In conventional supine MDCT, the gantry remains fixed while the patient moves through the scanning field with the examination table. In contrast, in the upright MDCT system, the gantry moves vertically along the patient’s body axis while the individual remains stationary [18, 19]. Because the gantry is large and rotates at high speed, any vibration or deviation from horizontal alignment during vertical motion may degrade image quality. To minimize such effects, the system incorporates several engineering solutions, including a high-speed motion control unit, an active horizontal-level compensation mechanism, and a rigid gantry structure designed to suppress motion-related artifacts [19]. For patient safety, the scanner includes pinch-prevention and contact-interlock systems [18, 19]. To maintain stability in the standing position, a supportive pole offers gentle posterior support, and a knee-height transparent acrylic barrier surrounds the patient to prevent falls (Fig. 1c).

**Table 1:** Comparison of key technical specifications and configurations between conventional and upright 320-detector-row CT systems

	Conventional CT	Upright CT
*Common specifications*		
Detector configuration	0.5 mm × 320 or 80	0.5 mm × 320 or 80
Maximum gantry rotation speed	0.275 s	0.275 s
Beam pitch	0.673, 0.813, 1.388 @ 80 detector mode	0.673, 0.813, 1.388 @ 80 detector mode
kVp	80, 100, 120, 135 kVp	80, 100, 120, 135 kVp
mA	10–600 mA	10–600 mA
Bore size	780 mm	780 mm
FOV (field of view)	500 mm	500 mm
Reconstruction	AIDR 3D, SEMAR	AIDR 3D, SEMAR
*System-specific features*		
Imaging position	Supine	Standing, Sitting
Maximum bed speed	160 mm/s	N.A.
Maximum gantry movement speed	N.A.	100 mm/s
Technique to prevent motion artifact due to gantry movement	N.A.	High-speed motion control system Horizontal compensation mechanism Highly rigid rotating structure
Installation space	–	Two-thirds compared to conventional CT

AIDR 3D = adaptive iterative dose reduction; N.A. = not applicable; SEMAR = single energy metal artifact reduction

### Evaluation of the shoulder and upper extremity

Upright MDCT enables precise three-dimensional (3D) assessment of the shoulder girdle and upper extremity under physiologic weight-bearing conditions, addressing the limitations of conventional supine MDCT, which restricts scapular motion and alters joint alignment.

**Fig. 2:**
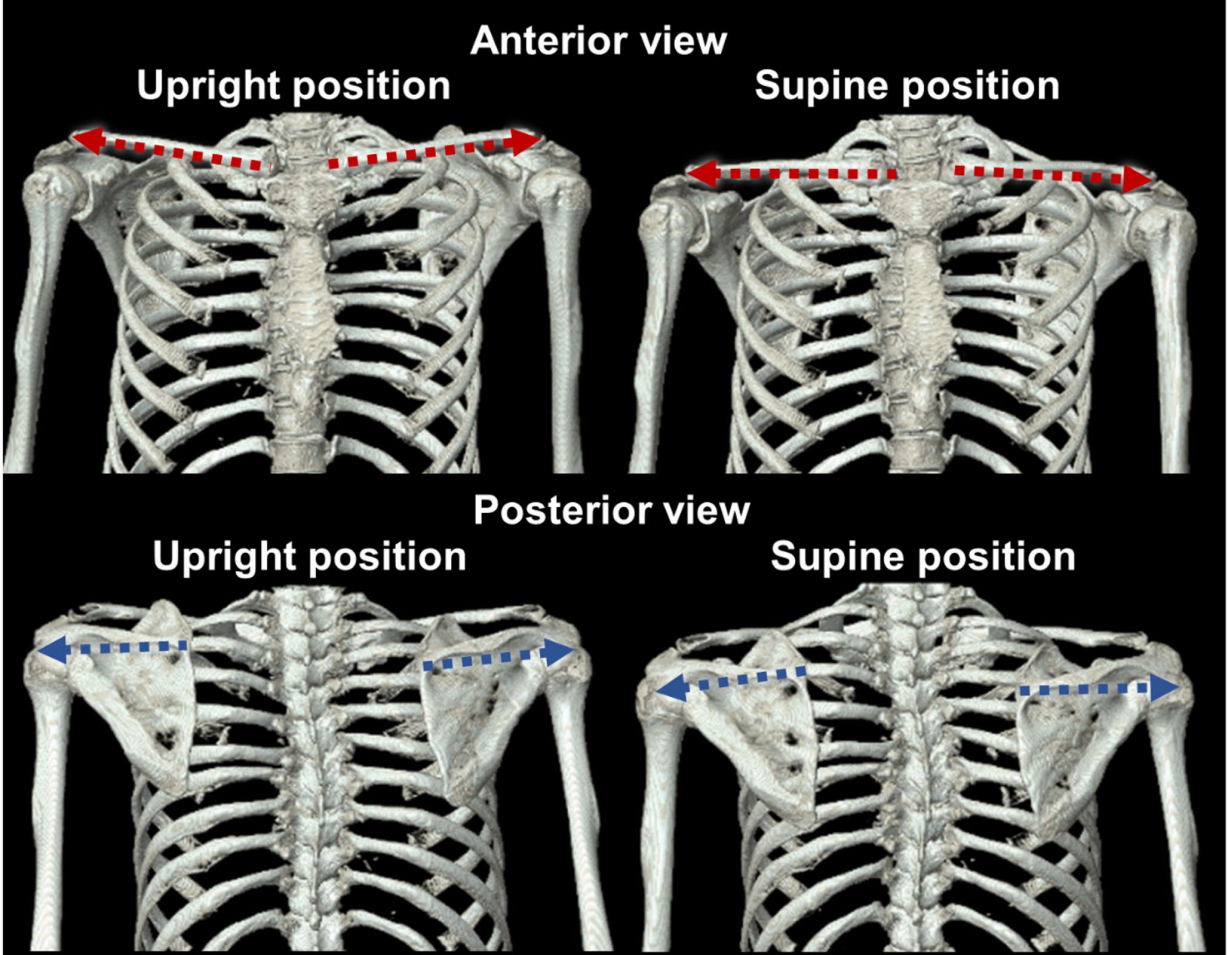
Volume-rendered CT images obtained in the supine (left) and upright (right) positions. Compared with the supine position, clavicular elevation (red dotted lines) and scapular upward rotation (blue dotted lines) are reduced in the upright position due to gravitational load

**Fig. 3:**
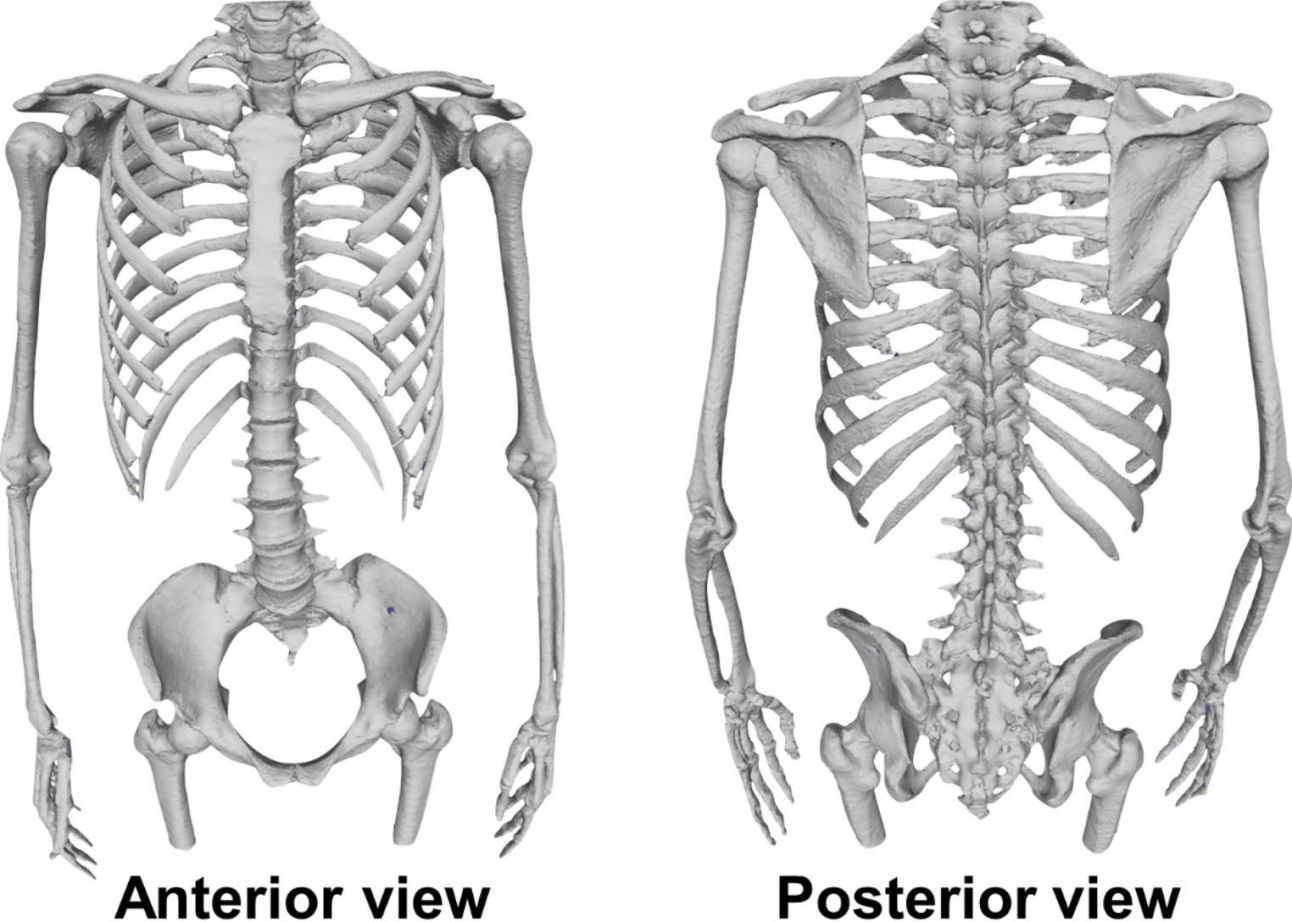
Three-dimensional surface models of the torso and upper extremities. These surface models were reconstructed from upright CT data and used to evaluate upper-extremity alignment in the standing position

Matsumura et al. [28] quantitatively compared 200 shoulders from 100 healthy volunteers between supine and standing positions and demonstrated significant gravitational effects on shoulder girdle alignment. In the standing position, the clavicle showed less elevation and greater retraction, whereas the scapula exhibited reduced upward rotation, anterior tilting, and internal rotation (Fig. 2). The clavicular center shifted inferiorly, posteriorly, and laterally, while the scapular center moved inferiorly, posteriorly, and medially, indicating position-dependent alignment changes due to gravity [28]. Building on this work, Yoshida et al. [29] examined 166 shoulders from 83 healthy volunteers and identified a difference in the acromiohumeral distance (AHD) between supine and standing positions, confirming that supine assessments tend to underestimate the AHD compared with standing evaluations [29]. Subsequently, Yoshida et al. [30] established normative 3D alignment values in 158 upper extremities from 79 healthy participants scanned in a standing neutral position (Fig. 3). Median scapulothoracic joint angles were 9.2° (interquartile range [IQR], 5.2°–12.5°) for upward rotation, 29.0° (IQR, 24.9°–33.3°) for internal rotation, and 7.9° (IQR, 4.3°–11.8°) for anterior tilt [30]. The glenohumeral joint showed median angles of 4.5° (IQR, 0.9°–7.8°) of abduction, 9.0° (IQR, 2.2°–19.0°) of internal rotation, and 0.3° (IQR, − 2.6°–3.1°) of extension. The elbow joint exhibited 9.8° (IQR, 6.9°–12.4°) of valgus, 90.2° (IQR, 79.6°–99.4°) of pronation, and 15.5° (IQR, 13.2°–18.1°) of flexion [30]. These normative data serve as a detailed baseline for evaluating upper-extremity alignment in the standing position.

**Fig. 4:**
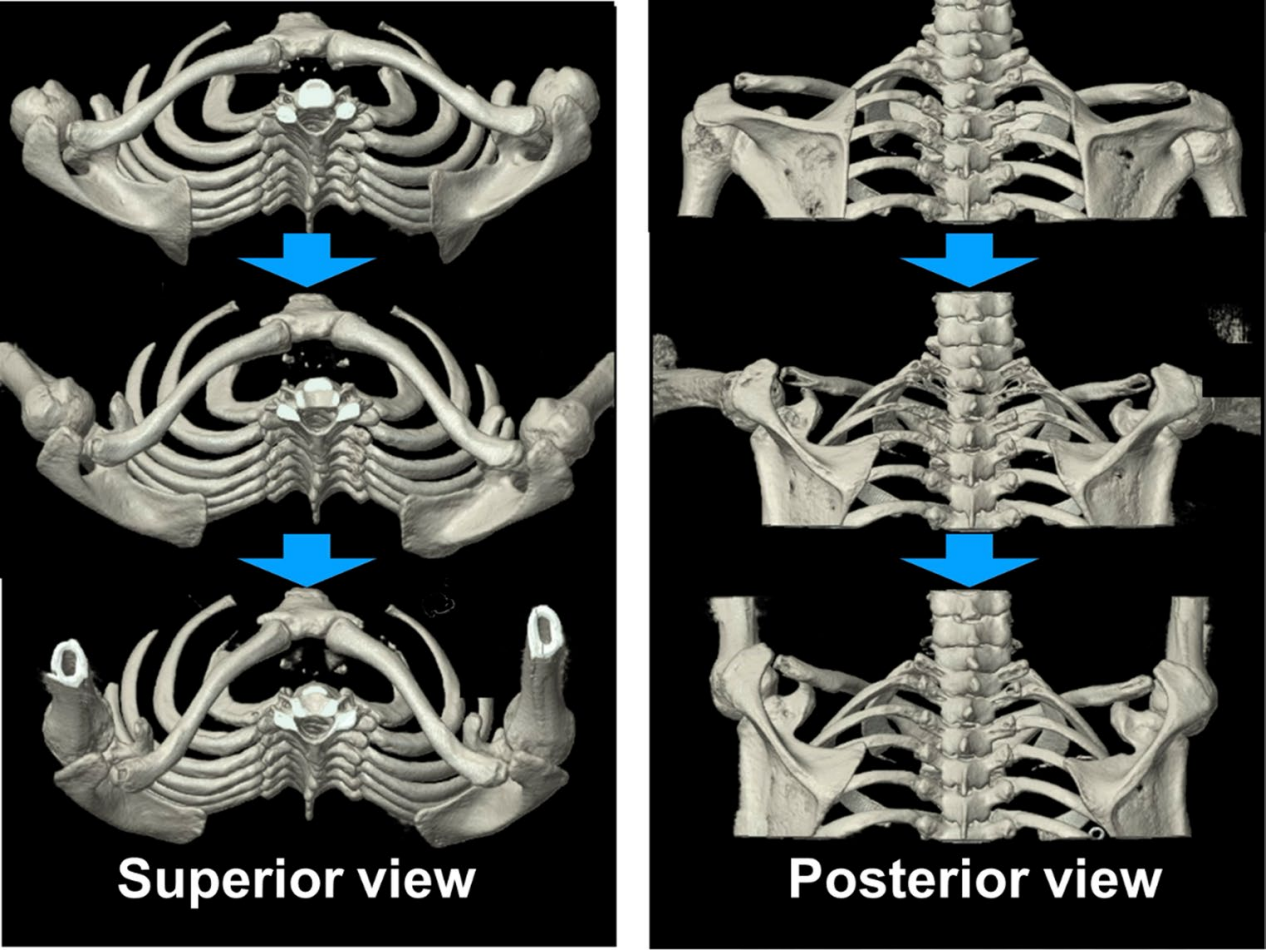
Upright four-dimensional CT images of shoulder motion. Superior (axial) and posterior (coronal) views demonstrate dynamic shoulder motion during arm elevation, enabling in vivo analysis of shoulder kinematics under physiologic load

**Fig. 5:**
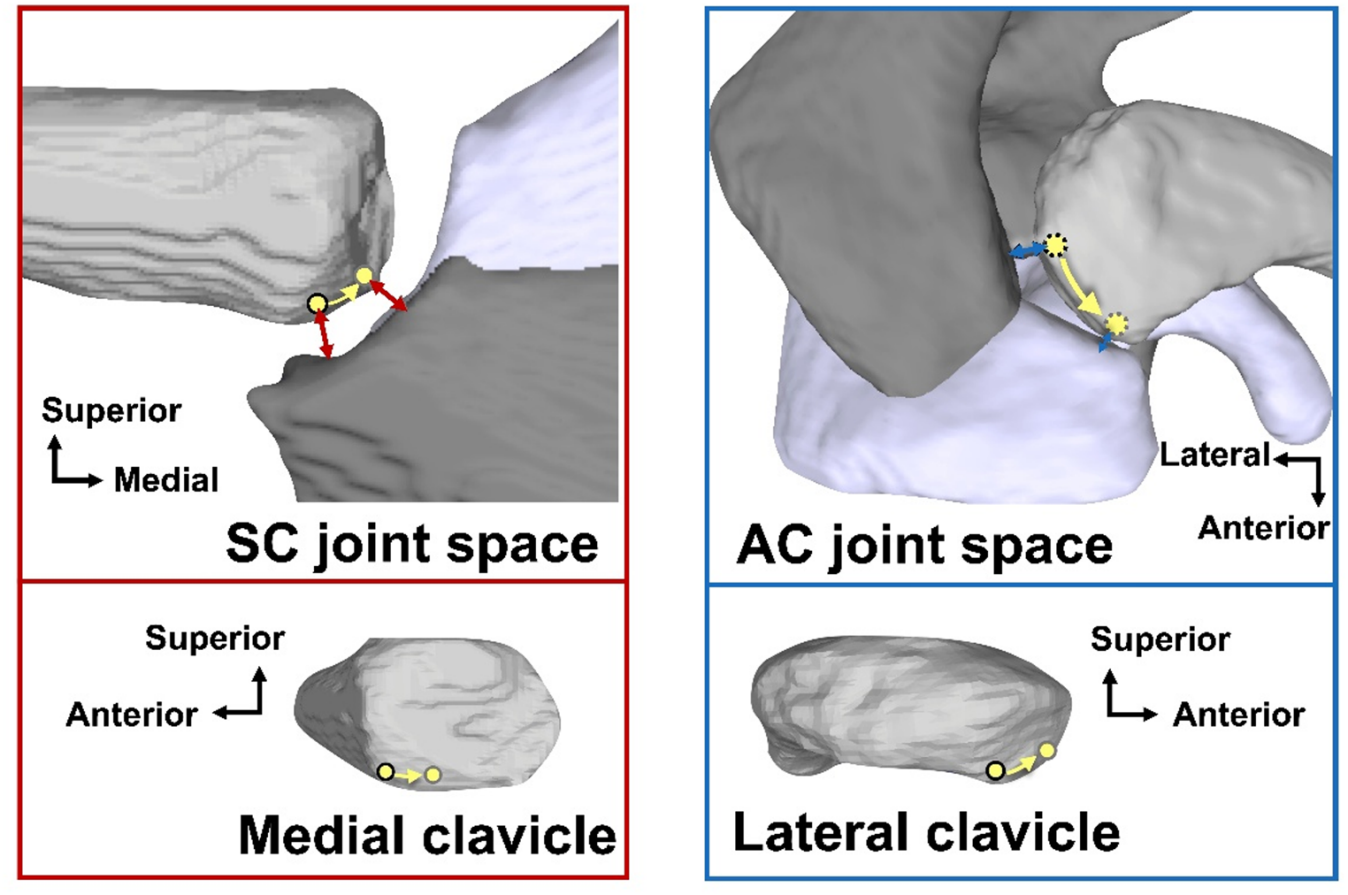
Three-dimensional representations of the sternoclavicular (SC) and acromioclavicular (AC) joint spaces during arm elevation. Arrows in the SC joint image indicate distances between the clavicle and sternum. Arrows in the AC joint image indicate distances between the clavicle and acromion and demonstrate progressive narrowing of the AC joint space during arm elevation. Yellow arrows show the movement of the closest contact points, and yellow circles mark their anteroinferior locations on the medial and lateral clavicle. Bones at the start of arm elevation are shown in gray, and those at the end are shown in white

**Fig. 6:**
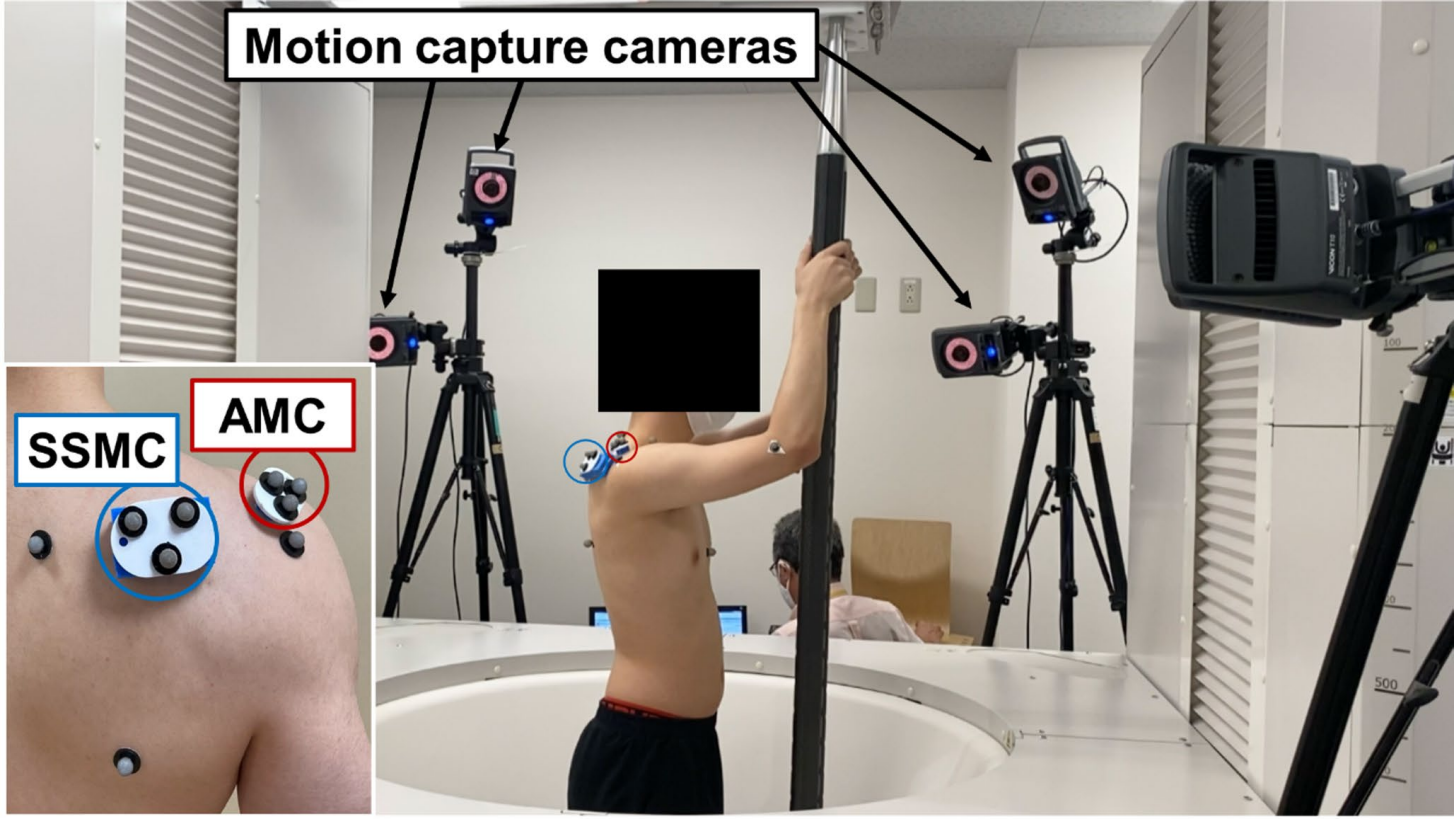
Shoulder elevation tracking using motion capture surrounding the upright CT scanner. An optical motion-capture system is positioned around the upright CT scanner, and motion capture cameras (black arrows) record shoulder motion. The acromion marker cluster (AMC; red circle) and scapular spinal marker cluster (SSMC; blue circle) are used to track scapular movement. Motion-capture data are compared with 4DCT measurements to validate the accuracy of the AMC and SSMC approaches

For dynamic evaluation, Yoshida et al. [31] validated upright four-dimensional CT (4DCT) as a method to track shoulder motion in vivo in 10 healthy male volunteers (20 shoulders) (Fig. 4 and Supplementary Video 1). During active humerothoracic elevation from 10° to 140°, the scapulothoracic joint showed upward rotation, internal rotation, and posterior tilting, while the glenohumeral joint demonstrated elevation, external rotation, and anterior-plane elevation [31]. The mean scapulohumeral rhythm, describing the relationship between glenohumeral and scapulothoracic motion during arm elevation and commonly expressed as the ratio of humeral elevation to scapular upward rotation, was 1.8 with 4DCT and 2.4 with optical motion capture, with significant differences at humerothoracic elevation ≥ 100°, supporting the superior precision of upright 4DCT at high arm positions [31]. Using the same 4DCT system, Yoshida et al. [32] characterized the kinematics of the sternoclavicular (SC) and acromioclavicular (AC) joints in 12 healthy volunteers (24 shoulders). During active “hands-up” motion, the clavicle elevated, retracted, and rotated posteriorly relative to the thorax, while the scapula rotated upward, internally, and posteriorly relative to the clavicle. The mean SC and AC joint-space distances were 2.2 ± 1.1 mm and 1.6 ± 0.9 mm, respectively [32]. The closest contact points were located at the anteroinferior regions of the medial and lateral clavicle. Significant angular changes occurred beyond 30°–50° of humerothoracic elevation, suggesting that repetitive impingement at these regions could contribute to osteoarthritis (Fig. 5) [32]. In a subsequent study, Yoshida et al. [33] compared two optical motion capture techniques—the acromion marker cluster (AMC) and scapular spinal marker cluster (SSMC)—against upright 4DCT in eight healthy male participants (16 shoulders) (Fig. 6). The AMC is a skin-mounted marker cluster attached to the acromion to track scapular motion while minimizing soft tissue artifacts. In contrast, the SSMC is attached to the mid-portion of the scapular spine, which is less influenced by deltoid contraction. Upright 4DCT provides bone-based kinematic measurements derived from dynamic CT imaging of the scapula, humerus, clavicle, and thorax and serves as the anatomical reference standard for comparison with optical motion capture. The mean angular differences between AMC and 4DCT were − 2.2° ± 7.5° for scapulothoracic upward rotation, 14.0° ± 7.4° for internal rotation, and 6.5° ± 7.5° for posterior tilt [33]. In contrast, SSMC showed smaller discrepancies (− 7.5° ± 7.7°, 2.0° ± 7.0°, and 2.3° ± 7.2°, respectively), although skin motion was larger (38.6 ± 5.8 mm with SSMC vs. 28.7 ± 4.0 mm with AMC) [33]. These findings indicate that AMC is more accurate for assessing upward rotation and elevation, whereas SSMC is preferable for internal rotation and posterior tilting [33]. These results do not imply that either marker cluster is inappropriate for general use; rather, they indicate that each method offers specific advantages depending on the axis of rotation evaluated. Clinically, this distinction is relevant because rotation angles of interest vary with shoulder activity, such as overhead motion, and selecting the marker cluster according to the primary axis of motion may improve the accuracy and reliability of scapular kinematic assessment.

Collectively, these studies suggest that upright MDCT provides a highly precise quantitative assessment of both static and dynamic parameters of the shoulder complex under physiologic loading conditions. This modality enables evaluation of posture-dependent variations in scapular and clavicular orientation that are not appreciable in the supine position and allows simultaneous visualization of SC and AC joint mechanics during motion. Therefore, upright MDCT may serve as a valuable approach for investigating biomechanical aspects of shoulder function and related pathologies, such as impingement and degenerative joint disease.

## Evaluation of the spine

Upright MDCT enables detailed three-dimensional (3D) evaluation of spinal morphology, alignment, and motion under physiologic loading conditions, providing clinically relevant insights that conventional supine imaging cannot offer. Its use in spinal assessment has shown that posture-dependent changes are frequent and often substantial, underscoring the importance of weight-bearing imaging in both research and clinical practice.

Fujita et al. [34] demonstrated that upright MDCT achieves excellent intra- and inter-observer reliability for spinopelvic measurements compared with spinal radiographs, and found that thoracic kyphosis measured on conventional radiographs could deviate by up to 6.6° from upright MDCT values, highlighting potential sources of error when assessing sagittal parameters with traditional methods.

Quantitative assessment of the lumbar intervertebral foramen has also revealed significant posture-dependent morphological changes. In a study of 30 patients with adult spinal deformity, > 5% changes in foraminal area or height (FH) were observed in approximately 30% of the 300 foramina examined when transitioning from the supine to the standing position [35]. These changes were most pronounced in the lower lumbar segments and were significantly more frequent on the concave side of scoliotic curves (*p* < 0.05) [35]. Narrowing of the intervertebral disc (cutoff > 0.05°) was identified as a significant risk factor for foraminal narrowing, suggesting a biomechanical mechanism underlying position-dependent radicular symptoms [35].

**Fig. 7:**
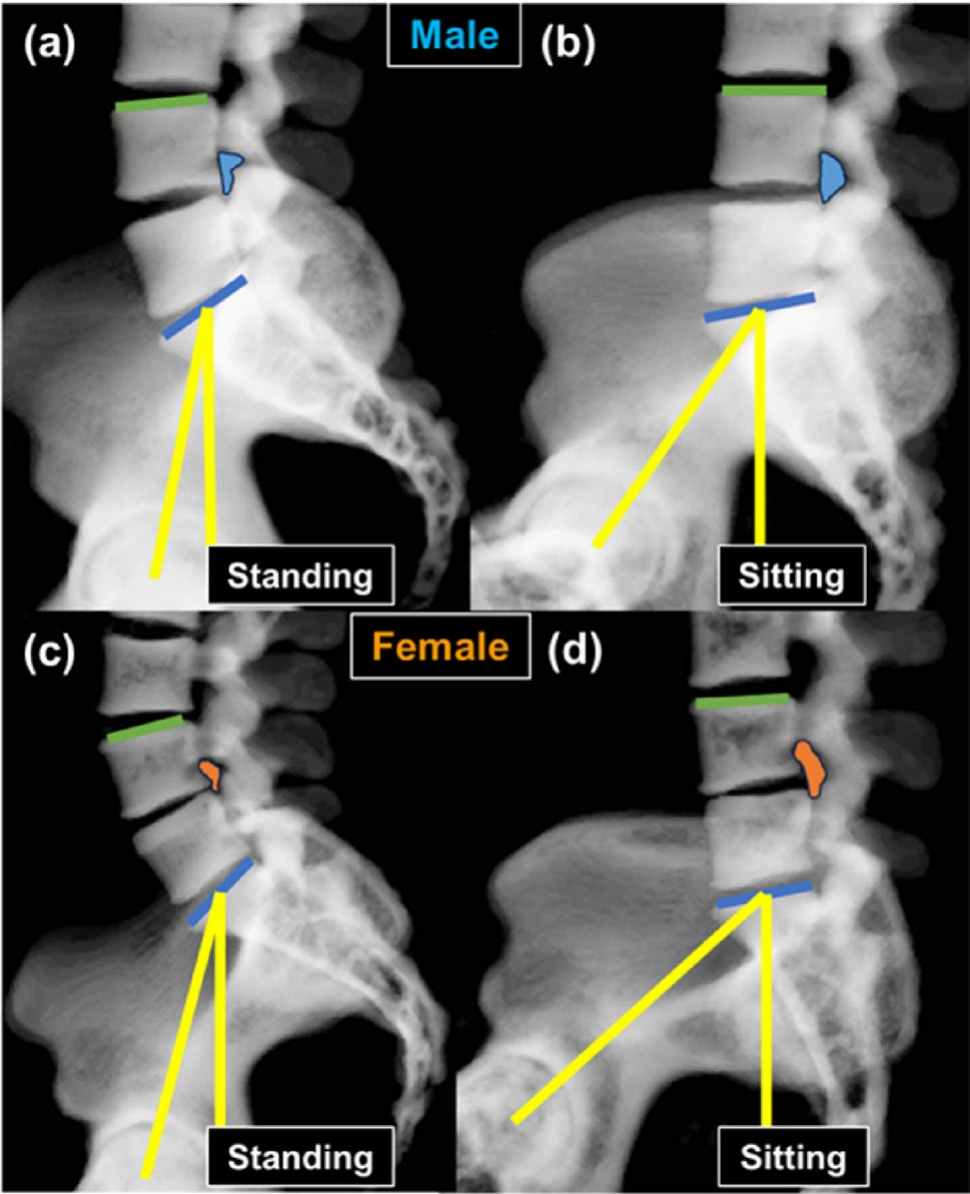
Representative cases demonstrating posture-related changes in spinal alignment and intervertebral foramen area in young adults. **a** Standing whole-spine CT of a 32-year-old male. **b** Sitting whole-spine CT of the same 32-year-old male. **c** Standing whole-spine CT of a 38-year-old female. **d** Sitting whole-spine CT of the same 38-year-old female. Solid yellow lines indicate pelvic tilt (PT), defined as the angle between the vertical axis and the line connecting the midpoint of the S1 endplate to the center of the femoral heads. The solid blue line on S1 indicates sacral slope (SS), and the angle between the green line on L4 and the blue line on S1 represents L4–S lumbar lordosis. The filled shape represents the bony boundary area (BBA) of the intervertebral foramen (blue in the male case; orange in the female case)

Upright MDCT has also enabled characterization of gender- and age-related differences in spinopelvic mobility. In a cohort of 49 healthy participants, Mizukoshi et al. [36] reported that females exhibited significantly larger changes in sacral slope (SS) and pelvic tilt (PT) when transitioning from standing to sitting (*p* = 0.044 and *p* = 0.038, respectively) and showed greater decreases in the L4–S lordotic angle among elderly participants (− 14.1° in females vs. −9.2° in males, *p* = 0.039) (Fig. 7). The SS, defined as the angle between the sacral endplate and the horizontal plane, and the PT, defined as the angle between the vertical line and the line connecting the hip axis to the midpoint of the sacral endplate, are fundamental indices for sagittal balance evaluation. PT reflects pelvic retroversion and serves as a key compensatory mechanism for spinal malalignment [37]. Regarding lumbar morphology, significant sex differences in FH changes during postural transitions were observed in the elderly population (1.8 mm in females vs. 0.4 mm in males, *p* = 0.04). In contrast, females exhibited significantly larger bony boundary area (BBA) values than males both in the entire cohort (23.8 mm² vs. 10.8 mm², *p* = 0.03) and among younger participants (35.6 mm² vs. 19.4 mm², *p* = 0.01) (Fig. 7). These differences may contribute to the greater prevalence of lumbar degenerative spondylolisthesis in females, especially at the L4/5 level [36].

**Fig. 8:**
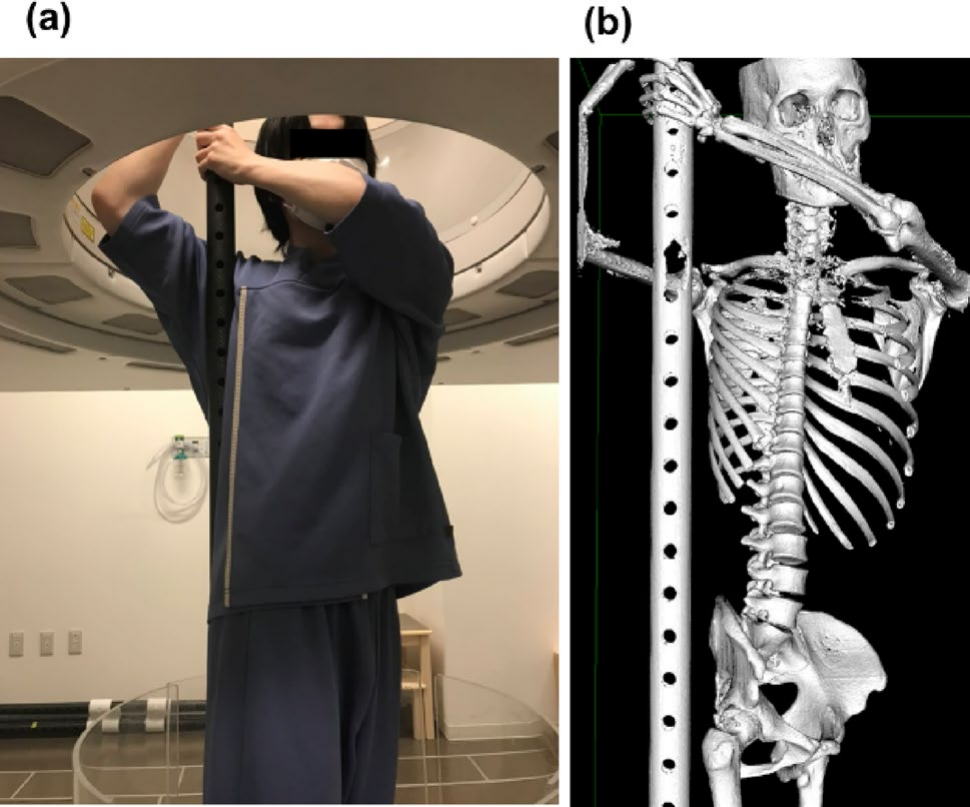
Methodology for acquiring maximal trunk rotation scans using upright CT. **a** Photograph of a healthy volunteer positioned inside the upright CT gantry, standing with both feet aligned on designated foot markers, performing maximal right trunk rotation while gripping a central stabilizing bar. **b** Corresponding three-dimensional (3D) volume-rendered image reconstructed from the scan obtained in this posture

Physiological rotational motion of the thoracolumbar spine has also been quantified with upright MDCT. In a study of 49 healthy volunteers, the lumbar spine exhibited a mean left rotation of − 1.3° ± 3.8° at L3 (*p* = 0.01), the lower thoracic spine rotated rightward by 1.9° ± 2.4° at T8 (*p* < 0.001), and the upper thoracic spine rotated leftward by − 2.6° ± 2.9° at T3 (*p* < 0.001) (Fig. 8) [38]. The p values from one-sample t-tests indicate that these vertebrae deviate significantly from a neutral 0° position even in natural standing posture. Notably, these rotational directions (left at L3, right at T8, and left at T3) align with characteristic rotation patterns observed in idiopathic scoliotic curves, suggesting that these physiological rotations may represent a biomechanical basis for common spinal deformity patterns [38]. During maximal trunk rotation, 76% of the motion occurred above T10, while only 16% occurred in the lumbar spine [38]. Total thoracolumbar rotation at T1 was significantly greater in females than in males (30.3° vs. 23.9°, *p* = 0.001) and in younger compared with older participants (29.2° vs. 25.0°, *p* = 0.028) [38].

The clinical relevance of upright MDCT extends beyond global alignment and segmental motion to include evaluation of neural and dural structures under load. Kawabata et al. [39] reported that, in 110 patients with lumbar degenerative disease, dural sac morphology showed significant posture-dependent changes: the anteroposterior diameter decreased at L2/3 and L4/5, while the transverse diameter increased at L1/2 and L2/3, and dural sac area increased at L1/2 but decreased at L4/5 in the standing position compared with supine MRI. These alterations were influenced by lumbar lordosis, indicating that upright imaging captures pathophysiologic features directly relevant to symptom generation [39]. This is clinically important because lumbar spinal stenosis symptoms are often provoked by upright posture and walking [40, 41].

**Fig. 9:**
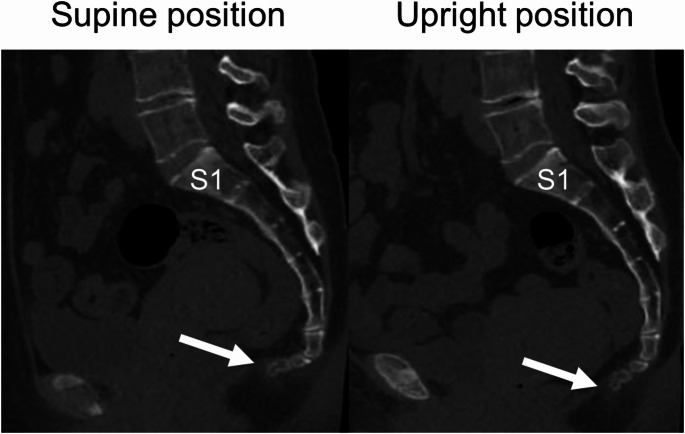
Sagittal CT images of the coccyx in a 60-year-old parous, healthy female volunteer in the supine (left) and upright (right) positions. In the supine position, the coccyx appears shorter and more curved, with anterior–superior displacement of the coccygeal tip (white arrow) compared with the upright position

At the caudal end of the spine, upright MDCT has also characterized posture-related changes in coccygeal alignment that may be linked to pelvic floor dysfunction and coccydynia. Yagi et al. [42] found that the coccygeal straight length increased from 35.7 ± 7.0 mm in the supine position to 37.8 ± 7.1 mm in standing, while the sacrococcygeal angle increased from 105.0° ± 12.5° to 115.0° ± 10.6°. The coccyx tip migrated 7.9 mm posteriorly and inferiorly when transitioning from the supine to the standing position, and this displacement correlated moderately with body mass index (*r* = 0.42, *p* = 0.0163) (Fig. 9) [42].

Overall, these findings indicate that upright MDCT offers a comprehensive approach for evaluating spinal biomechanics in a physiologically relevant state. It provides reproducible assessments of spinopelvic parameters, reveals posture-dependent variations in foraminal and dural morphology, characterizes sex- and age-related differences in spinal motion, and captures distal spinal dynamics that may contribute to pelvic floor pathology. Nevertheless, MRI remains the primary modality for evaluating soft tissue pathology in orthopedic imaging, whereas CT provides limited soft tissue contrast and involves ionizing radiation. In contrast to plain radiography, upright MDCT offers true three-dimensional quantitative assessment without structural superimposition. Furthermore, compared with upright MRI, typically performed at low magnetic field strength with limited spatial resolution and prolonged acquisition times [16, 17], upright MDCT enables rapid, high-resolution imaging of the entire skeletal system under physiological loading. In the spine, the key strength of upright MDCT lies in its ability to depict three-dimensional bony geometry and osseous morphology of the spinal canal and intervertebral foramina under load, which cannot be adequately captured by supine MRI or two-dimensional standing radiographs. While further studies are needed to clarify its clinical utility across diverse populations and disease states, upright MDCT appears to be a promising modality for advancing understanding of spinal function and improving the diagnostic evaluation of degenerative, deformity-related, and functional spinal disorders.

### Evaluation of the knee

Upright MDCT has significantly advanced the three-dimensional (3D) evaluation of knee morphology and biomechanics by enabling imaging under physiological weight-bearing conditions, thereby revealing alignment, rotational, and joint-surface characteristics that are not captured with conventional supine imaging. One major application has been the reappraisal of coronal plane alignment and joint line orientation, which have traditionally been assessed using two-dimensional (2D) radiography. Sasaki et al. [43] introduced the 3D tibial joint surface-floor angle, defined as the 3D angle between the tibial joint surface and the floor, and showed that the mean value measured on upright MDCT was 6.0 ± 3.6°, with no correlation to 2D joint line orientation. This finding highlighted the limitations of conventional measurements in capturing true joint line orientation [43]. Similarly, when the lateral distal femoral angle (LDFA) and the medial proximal tibial angle (MPTA) were measured using upright MDCT, significant discrepancies emerged. Briefly, LDFA is defined as the lateral angle between the femoral mechanical axis and the distal femoral joint line in the coronal plane, reflecting distal femoral coronal alignment. MPTA is defined as the medial angle between the tibial mechanical axis and the proximal tibial joint line, representing proximal tibial coronal alignment [44]. These angles are fundamental parameters for evaluating varus/valgus deformity and are clinically relevant for diagnosis and surgical planning of corrective osteotomy and total knee arthroplasty. In mild osteoarthritis (OA) (Kellgren–Lawrence [KL] 1 or 2), 2D-LDFA was 86.5 ± 1.8° compared with a 3D-LDFA of 85.0 ± 2.5° (*p* < 0.05), and in severe OA (KL 3 or 4), 2D-LDFA was 88.7 ± 2.5° versus 87.7 ± 3.2° (*p* < 0.05) [45]. Consequently, the agreement between 2D- and 3D-based coronal plane alignment of the knee classifications was only 48.5% overall, decreasing to 25.0% in mild OA [45].

**Fig. 10:**
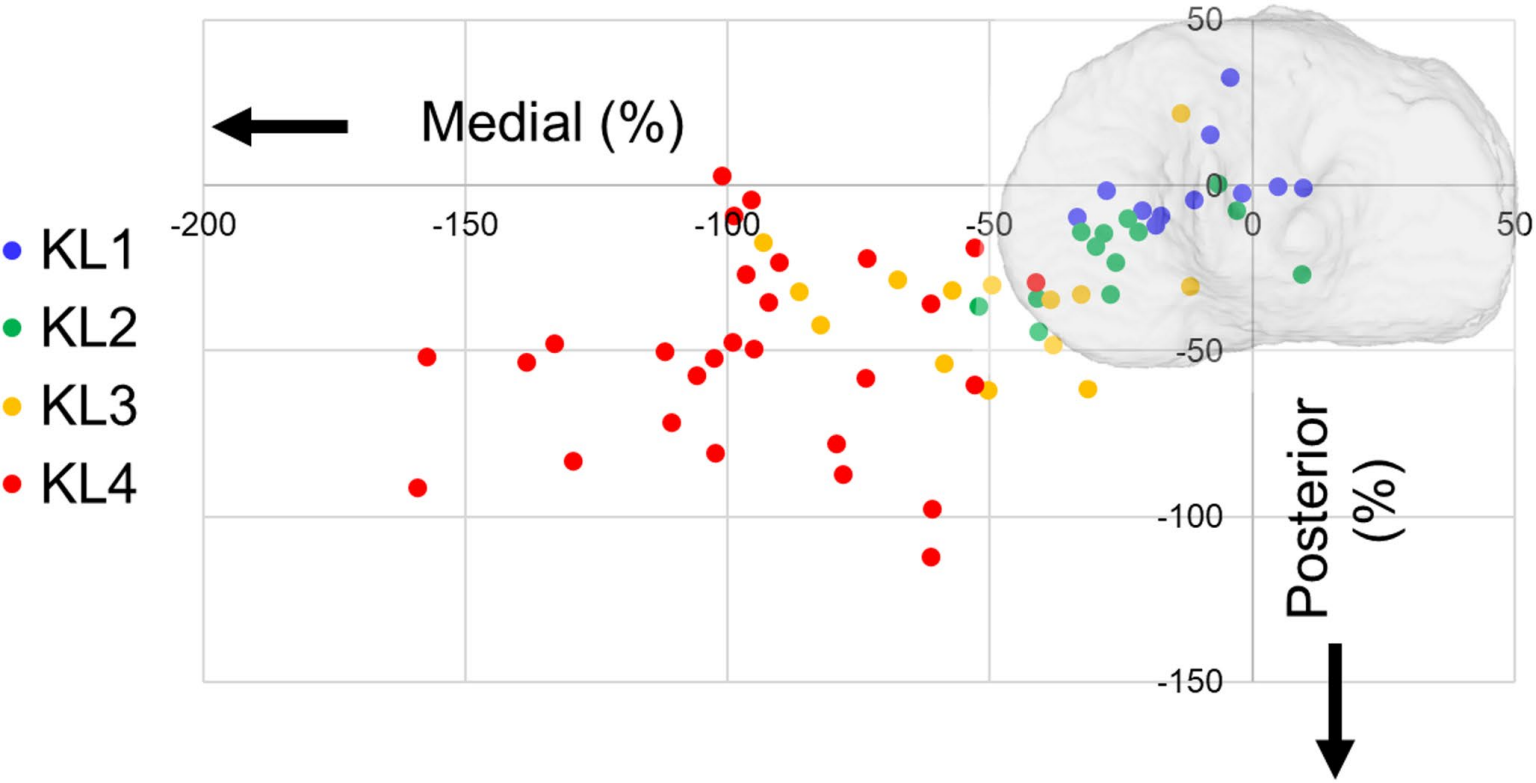
Scatterplot of the three-dimensional (3D) mechanical axis (MA). As the Kellgren–Lawrence (KL) grade increases, the 3D MA shifts medioposteriorly, indicating progressive varus and posterior deviation associated with advanced osteoarthritic changes. The 3D MA passes 5.3% medially and 7.1% posteriorly relative to the tibial plateau center in KL-1, shifting to 30.6% medially and 50.9% posteriorly in KL-3, and 56.7% medially and 92.3% posteriorly in KL-4

Upright MDCT has also revealed marked positional changes in the mechanical axis (MA) with disease progression. In a study of 66 varus OA knees, the 3D MA passed 5.3% medially and 7.1% posteriorly relative to the tibial plateau center in KL-1, shifting extra-articularly to 30.6% medially and 50.9% posteriorly in KL-3, and 56.7% medially and 92.3% posteriorly in KL-4 (Fig. 10) [43]. The mediolateral MA position correlated strongly with the femorotibial angle (*r* = − 0.85), and the anteroposterior position correlated with the knee flexion angle (*r* = − 0.93) [43].

**Fig. 11:**
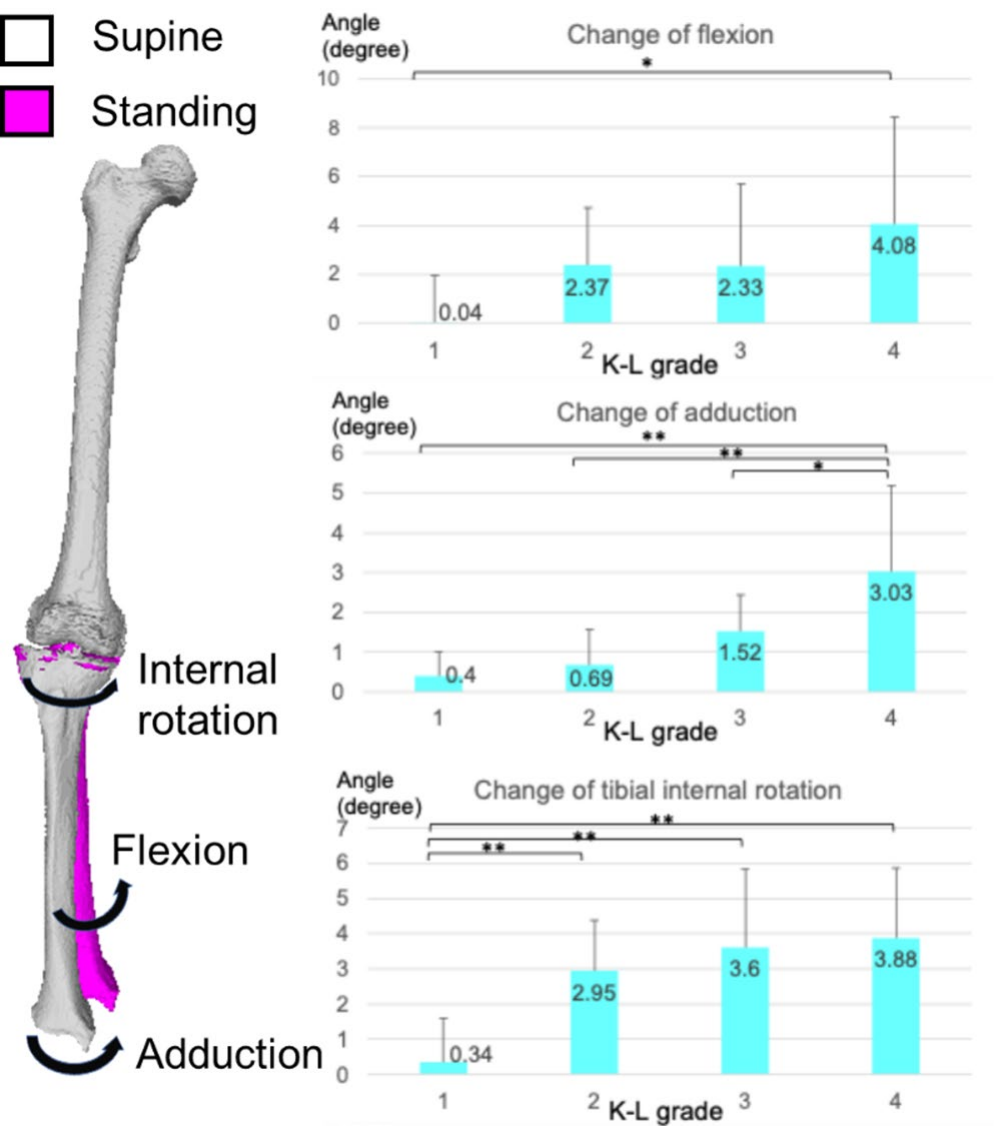
Comparison of supine and standing positions shows progressive increases in knee flexion, adduction, and tibial internal rotation with higher KL grade. Notably, tibial internal rotation demonstrates a marked increase between KL-1 and KL-2, preceding other angular changes. * indicates *p* < 0.05, ** indicates *p* < 0.01

**Fig. 12:**
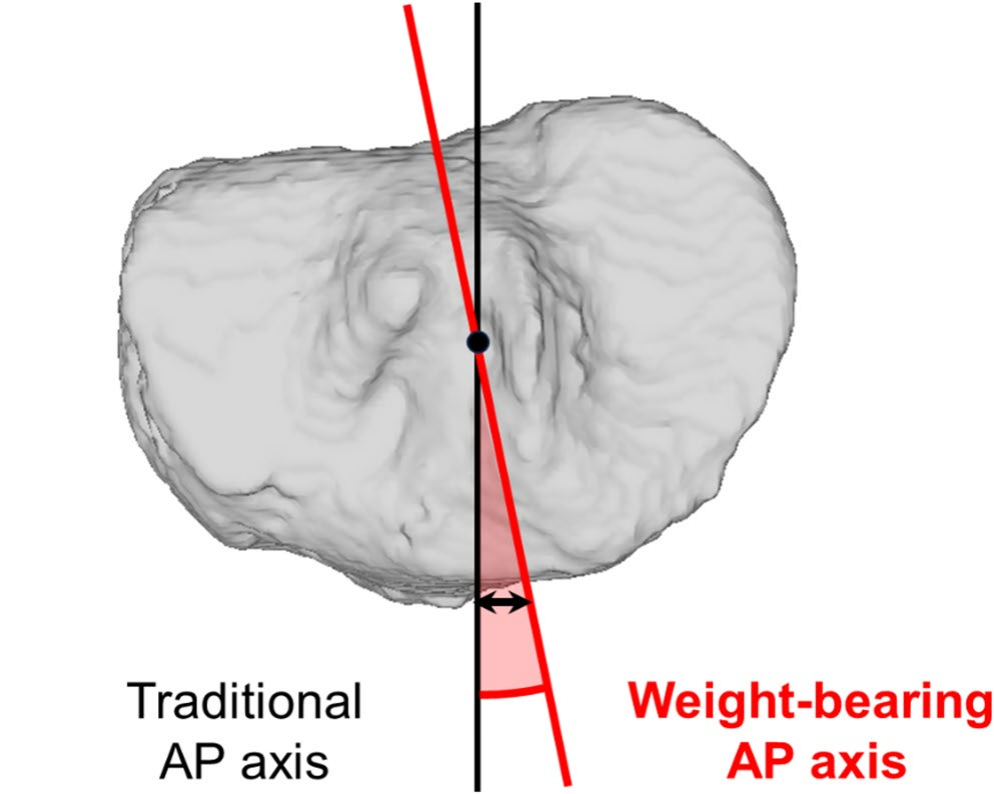
Upright anteroposterior (AP) axis of the tibia.Three-dimensional CT image illustrating the weight-bearing AP axis (red line), which is internally rotated by 7.4 ± 4.3° relative to the traditional non-weight-bearing AP axis (Akagi’s line; black line), with a mean displacement of 2.9 ± 1.6 mm at the tibial plateau edge (black arrow)

Dynamic changes induced by weight bearing represent another critical area of investigation. Kaneda et al. [46] reported that compared with supine conditions, standing induced significantly greater knee flexion, adduction, and tibial internal rotation. Notably, tibial internal rotation increased significantly between KL-1 and KL-2 knees, identifying this change as an early marker of OA progression (Fig. 11) [46]. Consistent with these results, Sasaki et al. [47] found that the weight-bearing anteroposterior (AP) axis of the tibia was internally rotated by 7.4 ± 4.3° relative to the traditional non-weight-bearing AP axis (Akagi’s line), with a mean displacement of 2.9 ± 1.6 mm at the tibial plateau edge, suggesting that conventional non-weight-bearing planning for total knee arthroplasty may underestimate physiologic rotational alignment (Fig. 12).

**Fig. 13:**
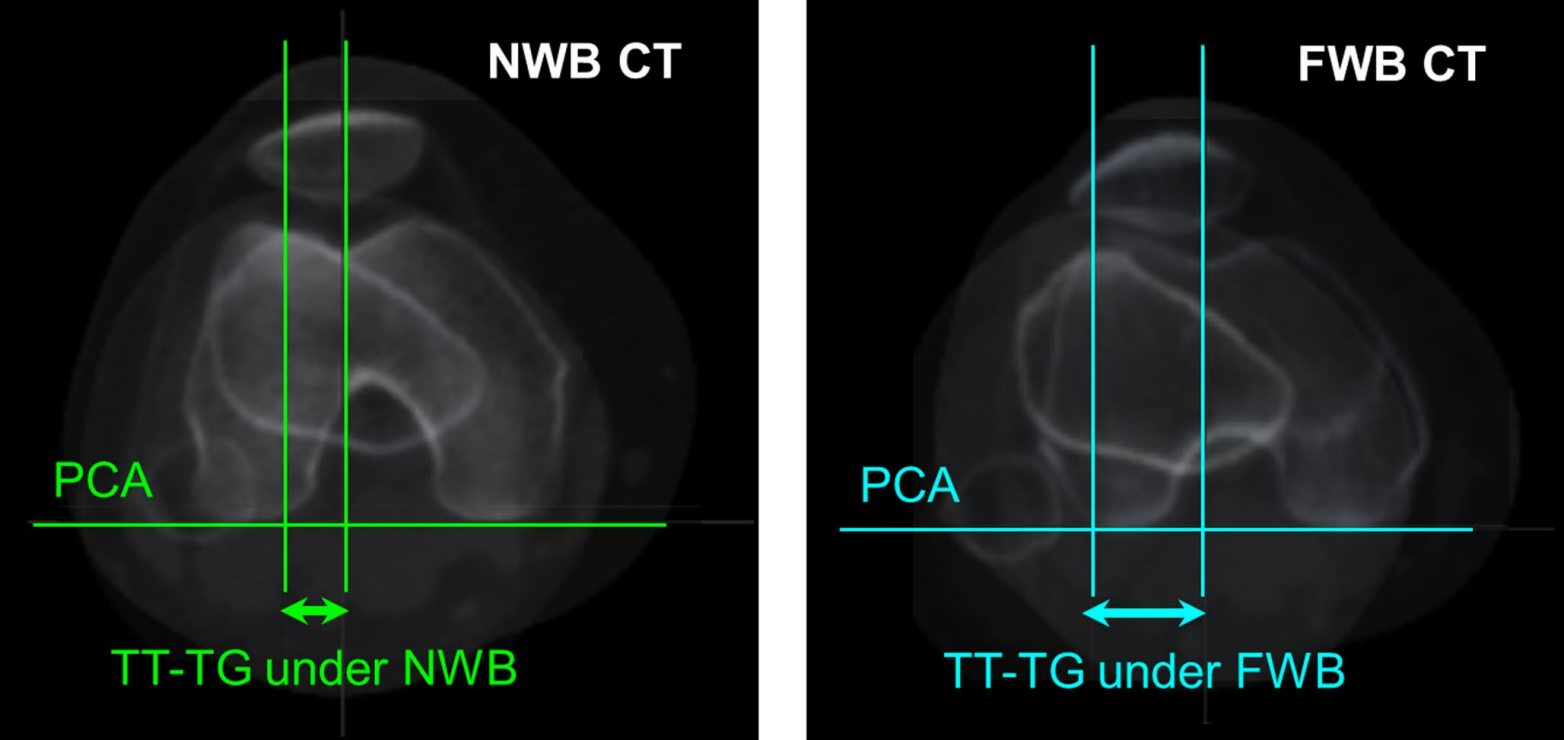
Axial CT comparison of the tibial tubercle–trochlear groove (TT–TG) distance under non-weight-bearing (NWB, left) and full-weight-bearing (FWB, right) conditions.The TT–TG distance increases from 12.3 ± 4.7 mm under NWB (green arrow) to 20.3 ± 3.9 mm under FWB (blue arrow) (*p* < 0.001), exceeding the conventional pathological threshold of 20 mm and potentially influencing indications for distal realignment in medial patellofemoral ligament reconstruction. PCA: posterior condylar axis

Patellofemoral alignment is also strongly influenced by load. In a cohort of 49 normal knees, the tibial tubercle–trochlear groove (TT–TG) distance increased significantly from 12.3 ± 4.7 mm under non-weight-bearing conditions to 20.3 ± 3.9 mm during full weight bearing (*p* < 0.001), exceeding the widely used pathological threshold of 20 mm and potentially altering indications for distal realignment in medial patellofemoral ligament reconstruction for recurrent patellar dislocation (Fig. 13) [48].

Upright MDCT has also characterized kinetic-chain interactions between the knee and distal lower-limb segments. Hakukawa et al. [49] reported significant correlations between varus deformity and ankle/hindfoot alignment, including talocrural internal rotation (*r* = 0.76) in KL I, subtalar eversion (*r* = 0.63) in KL II, and subtalar internal rotation (*r* = − 0.62) in KL III, underscoring compensatory realignment that occurs throughout the lower limb under pathological loading.

Taken together, these findings show that upright MDCT provides a physiologically relevant and quantitatively robust assessment of the knee, capturing load-dependent alterations in joint-line orientation, mechanical-axis trajectory, rotational kinematics, patellofemoral metrics, and lower-limb kinetic-chain behavior that conventional imaging does not depict. This more nuanced biomechanical understanding has important implications for diagnosis, disease monitoring, and preoperative planning, particularly for osteotomy, ligament reconstruction, and arthroplasty.

### Evaluation of load distribution and kinetic chain mechanics in the lower extremity from forefoot to hip

Extending analysis beyond the knee, whole-body upright MDCT now enables detailed investigation of lower-limb biomechanics in physiologic standing posture. This technology permits image acquisition during natural bipedal stance and provides a stable platform for evaluating unstable postures such as single-leg standing or standing on an inclined floor, thereby advancing the study of foot and ankle mechanics under true physiological loading.

**Fig. 14:**
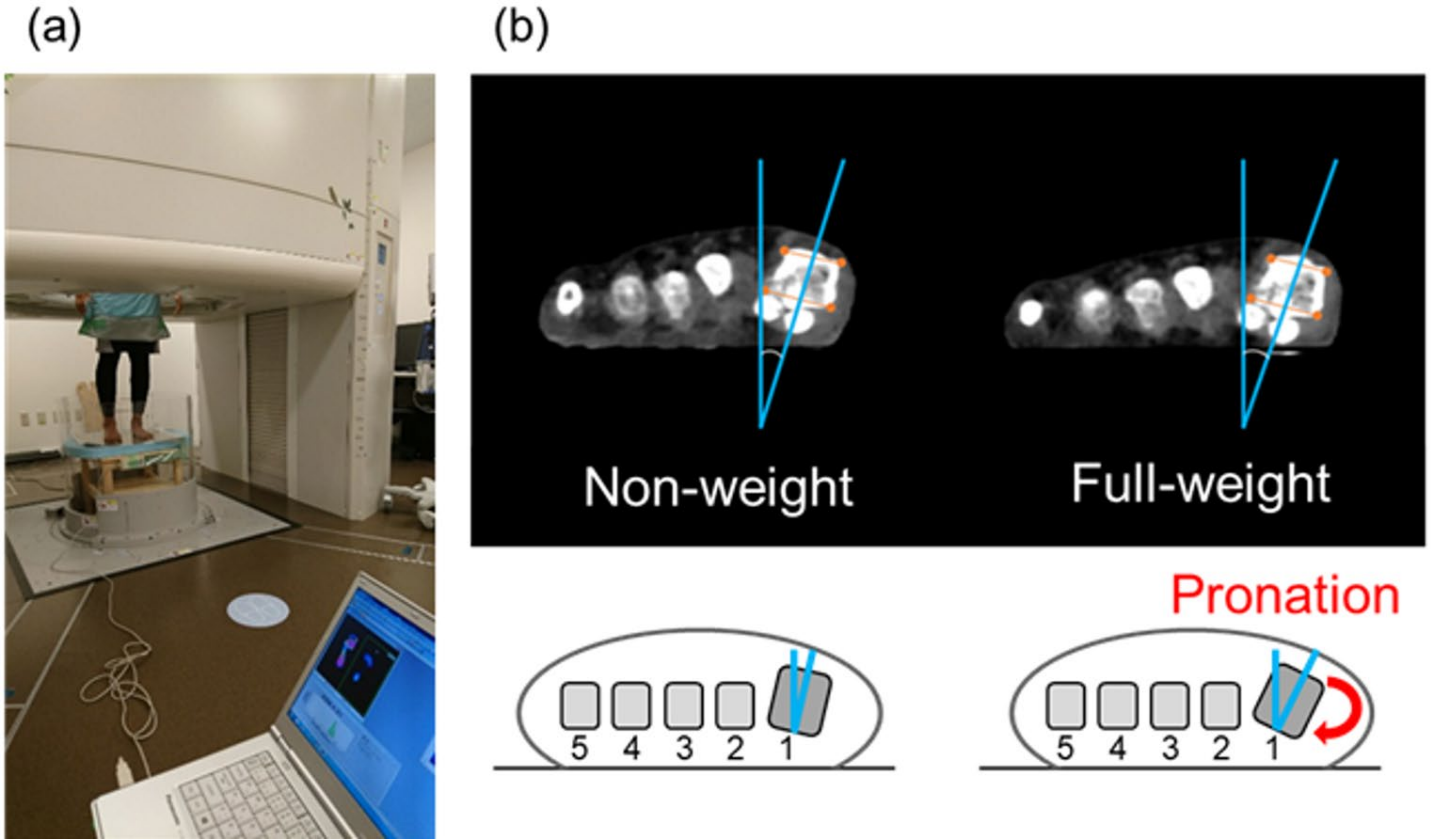
Quantification of first-metatarsal head pronation under weight-bearing. **a** Upright CT scans performed with plantar pressure monitoring to confirm load distribution during single-leg and bilateral weight-bearing. **b** Coronal-plane pronation angle of the first metatarsal head, demonstrating increased pronation under weight-bearing

**Fig. 15:**
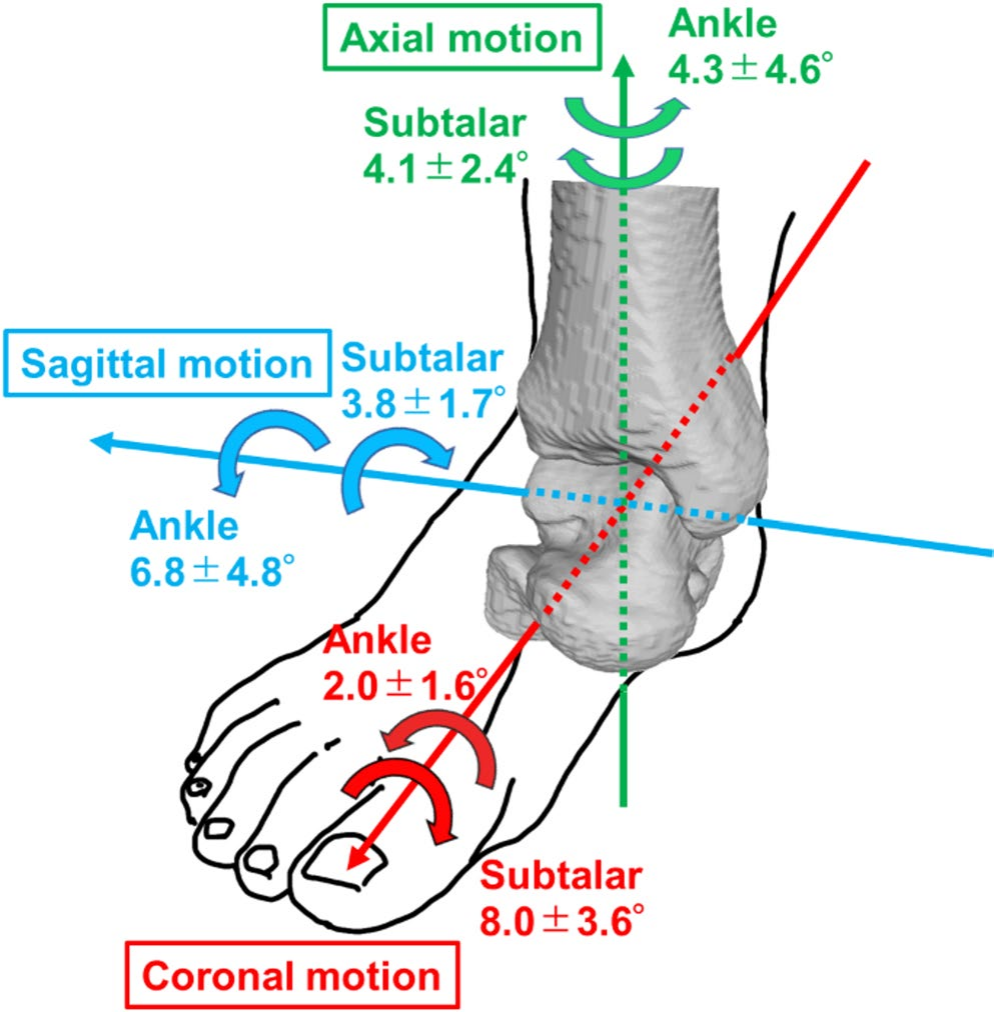
Weight-bearing 3D rotations at the tibiotalar and subtalar joints. Under full-weight-bearing, the talus shows plantarflexion (6.8 ± 4.8°), inversion (2.0 ± 1.6°), and internal rotation (4.3 ± 4.6°) relative to the tibia, while the calcaneus shows dorsiflexion (3.8 ± 1.7°), eversion (8.0 ± 3.6°), and external rotation (4.1 ± 2.4°) relative to the talus. These opposing rotations indicate counterbalancing motion with the talus acting as a central pivot, supporting joint congruency under vertical load. All axes are defined using the talar coordinate system

Ota et al. [50] reported that natural single-leg weight bearing significantly increased first metatarsal pronation compared with the non-weight-bearing condition (15.2° ± 5.4° vs. 12.5° ± 5.3°; *P* < 0.01), underscoring the role of forefoot torsion in accommodating load transfer and its potential implications for hallux valgus pathogenesis (Fig. 14). In addition, Kaneda et al. [51] conducted one of the first quantitative assessments of hindfoot kinematics under natural weight bearing, demonstrating joint-specific adaptations: under full single-leg load, the talus plantarflexed by 6.8 ± 4.8°, inverted by 2.0 ± 1.6°, and internally rotated by 4.3 ± 4.6° relative to the tibia, whereas the calcaneus dorsiflexed by 3.8 ± 1.7°, everted by 8.0 ± 3.6°, and externally rotated by 4.1 ± 2.4° relative to the talus (Fig. 15). These opposing rotational patterns highlight compensatory mechanics of the ankle and subtalar joints in maintaining alignment and stability under vertical load [51]. Building on these insights, Ogihara et al. [52] evaluated in vivo subtalar joint kinematics during weight bearing on 10° medially or laterally inclined floors in 15 healthy older adults (11 females, 4 males; mean age, 64.9 ± 5.0 years). The helical axis ran obliquely from the anterior–medial–dorsal to posterior–lateral–plantar direction, with inclination and deviation angles of 22.4° ± 9.6° and 35.2° ± 5.6° relative to the foot longitudinal axis, and 36.5° ± 10.7° and 12.0° ± 7.1° relative to the talar principal axis [52]. The mean rotation along this axis was 9.9° ± 3.8°, accompanied by minimal translation (0.5 ± 0.5 mm). These findings indicate that subtalar motion is more constrained under physiologic load than in non-weight-bearing conditions, emphasizing its stabilizing role during standing and the importance of upright imaging for characterizing hindfoot mechanics [52].

**Fig. 16:**
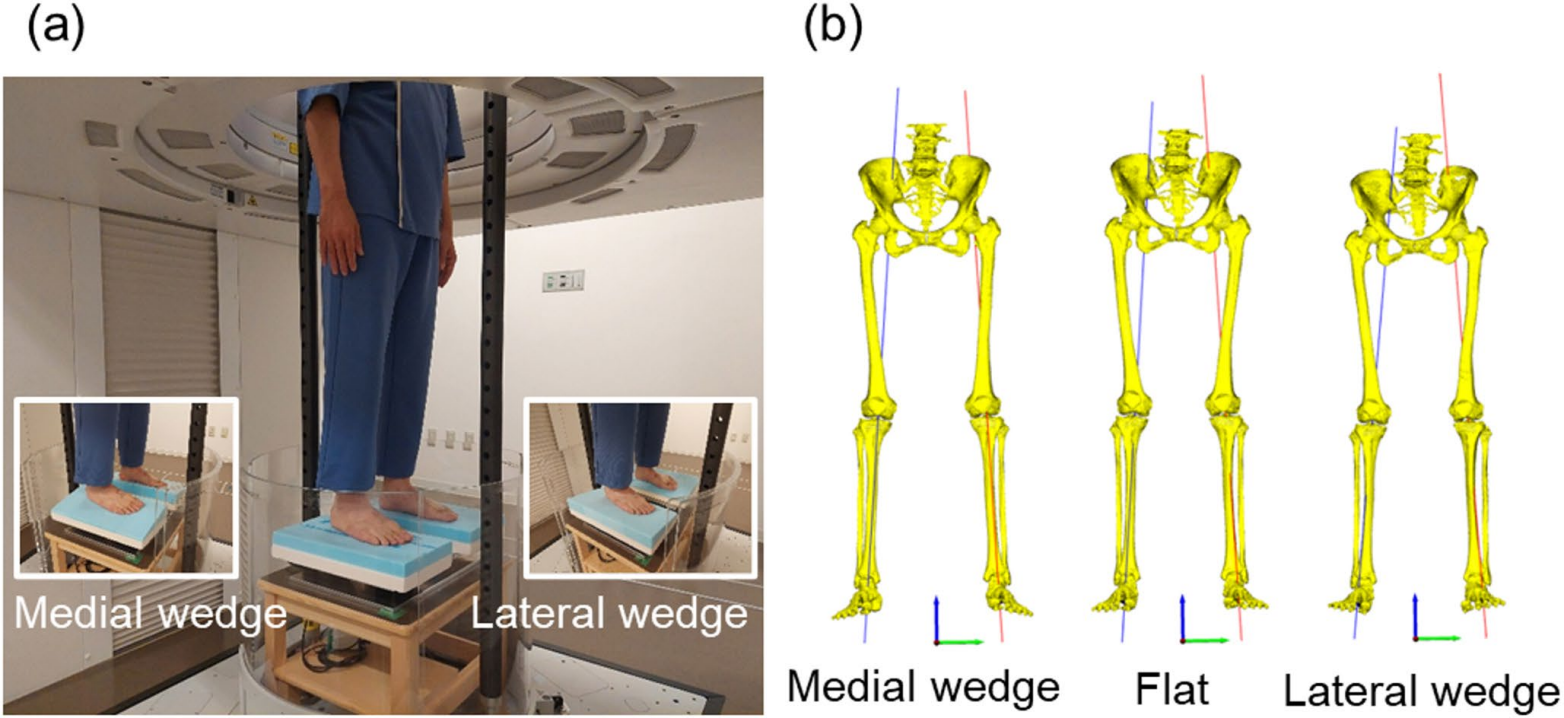
Floor condition–dependent changes in the ground reaction force (GRF) vector and lower limb alignment during standing. **a** Simultaneous upright MDCT and GRF acquisition using two six-axis force plates under three floor conditions: medial wedge (laterally inclined floor), flat, and lateral wedge (medially inclined floor). **b** Visualization of skeletal posture and GRF vectors across these conditions. GRF vectors shift closer to the hip, tibiotalar, and subtalar joints on the medial wedge and shift away on the lateral wedge, while the knee joint shows minimal change

Integration of joint-level biomechanics with ground reaction force (GRF) data has further advanced understanding of multi-joint interactions. Ito et al. [53] introduced a system that enables simultaneous quantification of three-dimensional skeletal posture and GRF vectors during quiet standing, showing that the actual force vector generally passes slightly medioposterior to the femoral head center rather than aligning with the conventional load-bearing axis. Building on this work, Seki et al. [54] examined how coronal wedge floors, defined as a 10° laterally inclined floor (medial wedge) and a 10° medially inclined floor (lateral wedge), influence lower-limb mechanics in older adults. In 15 participants (mean age, 64.9 ± 6.0 years), medial wedges induced hindfoot supination and hip abduction, whereas lateral wedges produced pronation and hip internal rotation [54]. Notably, the GRF vector consistently passed lateral to the tibiotalar and subtalar joints and medial to the hip, generating eversion and adduction moments, respectively [54]. These moments increased on a lateral wedge but diminished on a medial wedge, while knee moments remained minimal across all conditions (Fig. 16) [54]. This work provides the first three-dimensional quantification of GRF vectors relative to joint centers and underscores the clinical relevance of wedge orthotics for modulating load distribution across multiple joints [54].

**Fig. 17:**
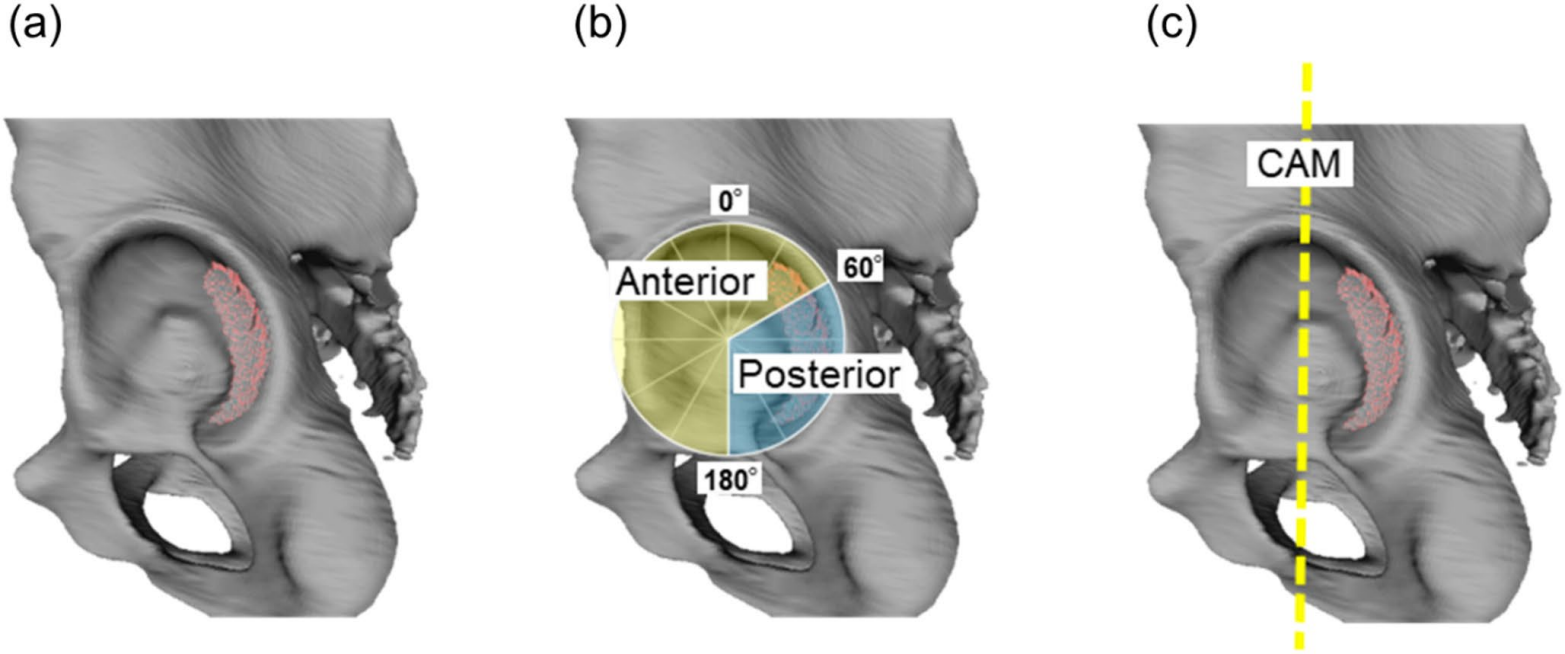
Hip joint proximity and sagittal balance during standing. **a** Upright CT depiction of the hip joint proximity area, defined as the acetabular surfaces within 1 mm of the femoral head. **b** Classification into anterior and posterior proximity groups based on the location of the closest contact area. **c** Comparison of sagittal balance parameters between groups, using a vertical line passing through the center of the acoustic meatus (CAM; yellow dotted line), which represents the gravity line

At the proximal end of the kinetic chain, Kikuchi et al. [55] used upright CT to show that the sagittal distribution of the hip joint proximity area, defined as regions of < 1 mm surface-to-surface distance between of the acetabulum and femoral head, correlates with global sagittal balance. Individuals with anterior proximity demonstrated significantly higher sagittal vertical axis and T1 spinopelvic inclination, suggesting anterior spinal alignment and a forward shift of the load line, whereas those with posterior proximity exhibited posterior gravity-line deviation relative to the hip center (Fig. 17) [55]. These findings indicate that upright MDCT can detect subtle variations in hip load distribution associated with spinopelvic alignment, which may be relevant to the pathomechanics of hip osteoarthritis and femoroacetabular impingement [55].

Taken together, these studies highlight the capability of upright MDCT to delineate load-dependent adaptations throughout the lower limb. By allowing simultaneous structural and kinetic evaluation in a functional posture, upright MDCT may support investigation of musculoskeletal pathophysiology, improve optimization of orthotic strategies, and inform surgical planning in conditions such as hallux valgus, ankle instability, hip osteoarthritis, and sagittal imbalance.

### Evaluation of the thoracic cage morphology

**Fig. 18:**
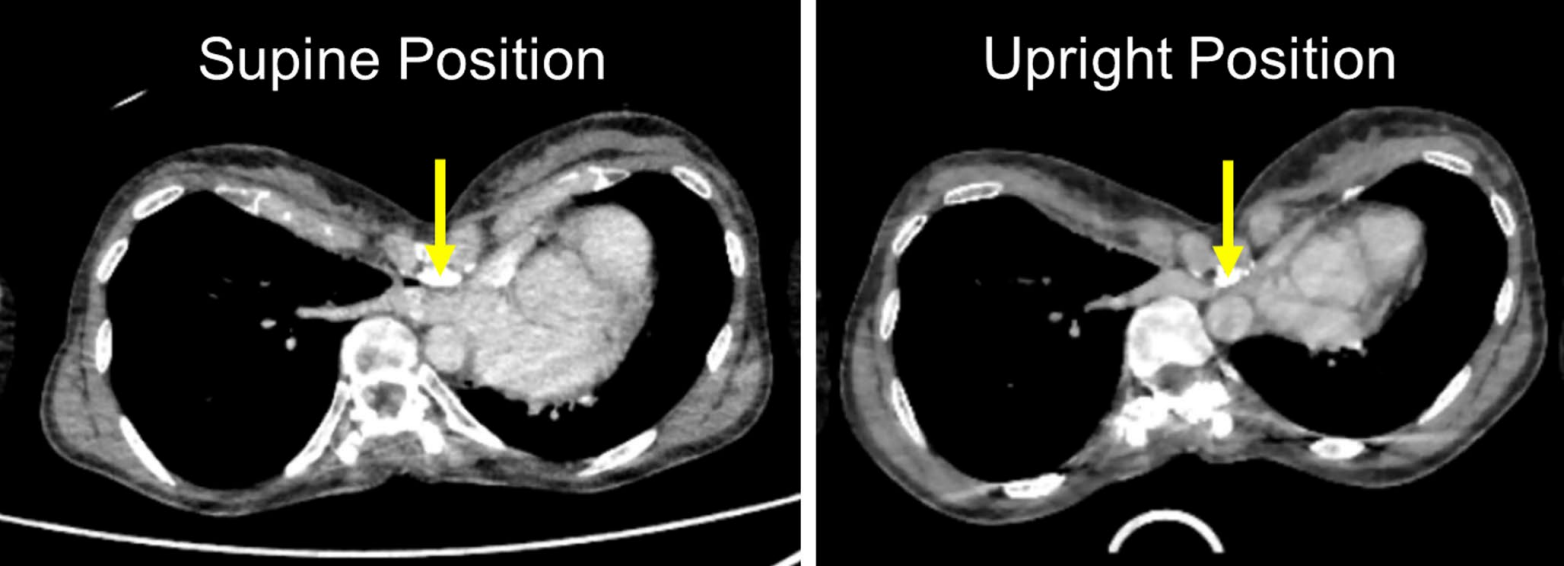
Horizontal CT images at the level of maximal chest depression in a 49-year-old patient with pectus excavatum presenting with upright-worsened dyspnea. Images were obtained during deep inspiration in the supine (left) and upright (right) positions. The right inferior pulmonary vein (yellow arrow) is markedly compressed in the upright position (right)

Posture-dependent thoracic cage morphology has important clinical implications, particularly in pectus excavatum (PE). A prospective study using both upright and supine MDCT in 21 patients with PE and 35 healthy volunteers showed that the minimum anterior–posterior thoracic diameter was significantly smaller in the upright position (4.5 ± 1.9 cm vs. 5.1 ± 1.9 cm, *P* < 0.001), whereas the Haller index was higher (10.1 ± 16.7 vs. 6.4 ± 4.4, *P* < 0.001) in patients with PE [56]. Similar, but less pronounced, changes in the minimum anterior–posterior thoracic diameter were observed in healthy volunteers (8.8 ± 1.9 cm vs. 9.2 ± 1.9 cm, *P* < 0.001) [56]. These changes were accompanied by an anterior shift of the lower thoracic vertebrae, with the distance between T6 and T12 plumb lines increasing significantly in the upright position (2.4 ± 1.0 cm vs. 0.8 ± 0.7 cm, *P* < 0.001) in patients with PE [56]. This vertebral displacement leads to narrowing of the thoracic cavity and may exacerbate cardiovascular compression in severe PE. In one patient, upright MDCT uniquely demonstrated right inferior pulmonary vein compression associated with dyspnea (Fig. 18), which resolved following surgical repair [56]. These findings show that upright MDCT captures posture-related thoracic cage changes that may be underestimated on conventional supine imaging, underscoring its clinical value in evaluating chest wall deformities under physiologically relevant conditions.

### Evaluation of skeletal muscle morphology under physiological loading

**Fig. 19:**
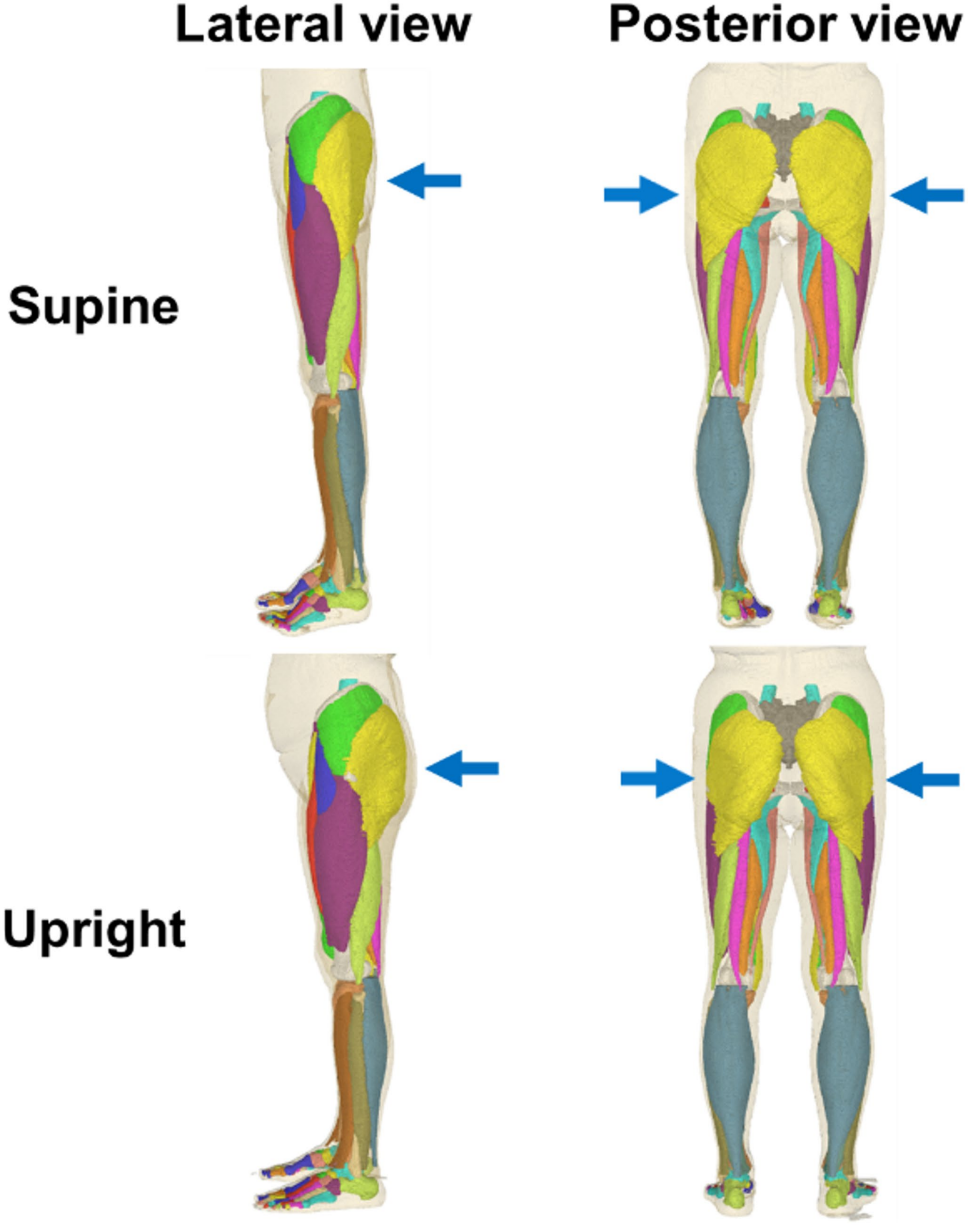
Volume-rendered images of automatically segmented skeletal muscles of the pelvis and lower extremities derived from supine (top) and upright (bottom) CT images of the same 48-year-old female healthy volunteer. Muscle morphology differs markedly between the two positions, with particularly pronounced differences in the gluteal region (arrows). The yellow muscles indicated by arrows represent the gluteus maximus

One advantage of the presented upright MDCT system over weight-bearing cone-beam CT is its superior soft-tissue contrast [13–15, 18, 19], enabling more precise and quantitative assessment of skeletal muscle. Upright MDCT allows three-dimensional evaluation of muscle morphology under physiologically relevant weight-bearing conditions that reflect daily posture. This is clinically important because age-related changes and the degree of muscle atrophy vary substantially among individuals and across different anatomical regions within the same individual [57–59]. Furthermore, muscle shape and configuration differ markedly between supine and upright positions owing to gravitational loading and changes in muscle tone and joint alignment (Fig. 19), indicating that supine imaging alone may not adequately represent functional muscle morphology. Recent advances in deep learning–based image analysis have enabled highly accurate, fully automated measurement of individual muscle volume and density from CT images [60]. If implemented in health screening centers, upright MDCT would enable longitudinal tracking of annual changes in muscle volume, morphology, and quality under physiologically relevant conditions. Such data could facilitate identification of region-specific muscle loss and support personalized exercise recommendations. Accordingly, upright MDCT has the potential to contribute to frailty and sarcopenia prevention in a super-aging society by enabling early detection of muscle deterioration and supporting individualized interventions guided by objective upright imaging findings.

### Limitations

Despite its considerable potential, upright MDCT has some limitations. First, findings in the existing literature and evaluations of clinical throughput, installation space, and radiation exposure are largely derived from single-center studies, which remain a concern. Strengthening the evidence base through well-designed multicenter collaborative studies is therefore warranted to establish broader clinical validity and generalizability. Second, the 320-detector upright MDCT was manufactured in limited numbers, with only five units produced, and has therefore not been widely disseminated in routine clinical practice. Nevertheless, recognition of the importance of evaluating individuals under physiologically relevant conditions is increasing [19, 20]. Recently, a commercially available multi-position MDCT system capable of supine, sitting, and standing imaging using a single scanner has been developed. Deployment of such systems is planned in Japan and other countries, and their clinical use is expected to expand globally, which may facilitate the dissemination of physiologically relevant CT imaging. Third, although upright MDCT can be performed in standing and sitting positions and includes safety-support devices to stabilize posture, examinations may remain challenging for patients with difficulty maintaining an upright position.

## Conclusion

Upright MDCT enables high-resolution, whole-body, three-dimensional imaging under physiologically relevant weight-bearing conditions, thereby overcoming the inherent limitations of conventional supine imaging. By depicting posture-dependent anatomical and biomechanical changes throughout the musculoskeletal system, it offers new insights into joint alignment, load distribution, and functional adaptation. As noted above, quantitative reference values for surgical indications on CT, which have traditionally been based on supine imaging, may in the future be redefined using upright imaging that more accurately reflects physiological loading conditions. These capabilities underscore the potential of upright MDCT to enhance diagnostic accuracy, deepen understanding of musculoskeletal pathophysiology, and support more individualized clinical decision-making and surgical planning. Accordingly, upright MDCT is poised to become a valuable modality in advancing orthopedic diagnostics and musculoskeletal research. 

## Supplementary Information

Below is the link to the electronic supplementary material.

Supplementary file1 (MP4 5584 KB)

## Data Availability

As this article is a narrative review based on previously published literature, no new data were generated or analyzed; therefore, data sharing is not applicable.
